# Dispersive effects and focused biodistribution of recombinant human hyaluronidase PH20: A locally acting and transiently active permeation enhancer

**DOI:** 10.1371/journal.pone.0254765

**Published:** 2021-07-22

**Authors:** David W. Kang, Beate Bittner, Barry J. Sugarman, Monica L. Zepeda, Marie A. Printz

**Affiliations:** 1 Halozyme Therapeutics, Inc., San Diego, CA, United States of America; 2 F. Hoffmann-La Roche Ltd., Basel, Switzerland; 3 Formerly with Halozyme Therapeutics, Inc., San Diego, CA, United States of America; ISF College of Pharmacy, INDIA

## Abstract

**Background:**

Recombinant human hyaluronidase PH20 (rHuPH20) facilitates the dispersion and absorption of subcutaneously administered therapeutic agents. This study aimed to characterize the transient, local action of rHuPH20 in the subcutaneous (SC) space using focused biodistribution and dye dispersion studies conducted in mice.

**Materials and methods:**

To evaluate the biodistribution of rHuPH20, mice were intradermally administered rHuPH20 (80 U). The enzymatic activity of rHuPH20 was analyzed in the skin, lymph nodes, and plasma. Animal model sensitivity was determined by intravenous administration of rHuPH20 (80 U) to the tail vein. To evaluate local dispersion, mice received an intradermal injection of rHuPH20 followed by an intradermal injection of Trypan Blue dye at a contralateral site 45 minutes later. Dye dispersion was measured using a digital caliper.

**Results:**

After intradermal rHuPH20 injection, enzymatic activity was detected within the skin near the injection site with levels decreasing rapidly after 15 minutes. There was no clear evidence of systemic exposure after administration of rHuPH20, and no discernible rHuPH20 activity was observed in lymph or plasma as a function of time after dosing. In the dye dispersion study, delivery of rHuPH20 at one site did not impact dye dispersion at a distal skin site.

**Conclusion:**

These observations support the classification of rHuPH20 as a transiently active and locally acting permeation enhancer.

## Introduction

Biotherapeutics, such as immunoglobins or monoclonal antibodies, typically require subcutaneous (SC) or intravenous (IV) administration, as oral administration is constrained by degradation, proteolytic digestion, and poor absorption in the gastrointestinal tract [1,2]. SC drug delivery is generally more convenient than IV delivery and can be associated with reductions in treatment burden and healthcare resources compared with conventional IV infusion [3–9]. However, SC administration of larger volumes typically requires more frequent dosing compared with IV administration, as well as multiple injection sites or slow flow rates, which can result in less reproducible systemic exposure to therapies and reduced patient convenience [3,10–12]. With SC administration, therapeutics are delivered to the SC extracellular space before dispersing locally and being absorbed by lymphatic or blood capillaries [2]. The molecular size of therapeutics is a major factor affecting uptake from the SC site; larger molecules (10–100 nm) are primarily absorbed by the lymphatic capillaries whereas smaller molecules (>10 nm) are absorbed by the blood capillaries [13].

The extracellular matrix impedes the rapid dispersion and absorption of larger fluid volumes following SC administration. The resistance to bulk fluid flow is mainly attributed to the large, naturally occurring glycosaminoglycan, hyaluronan (HA) in the SC space [2,11]. HA associates with water to form a gel-like substance in the interstitial matrix [2,11]. In standard situations, when therapeutic proteins are administered into the SC space, they slowly diffuse through the gel-like, hydrated HA interstitium [13]. As part of normal homeostatic processes, HA is depolymerized by hyaluronidases into smaller fragments that are eliminated through the lymphatic or circulatory systems, and resynthesized by HA synthases in the tissues [14,15]. Unlike the HA fragments, larger biotherapeutics such as antibodies (typically ~150 kDa in size) cannot be absorbed by blood capillaries [14,16]. With a tissue half-life ranging from several hours to several days [17], HA is a suitable target for modification to enable SC injection of large fluid volumes [11]. The rapid depolymerization of HA by hyaluronidase decreases the viscosity of the SC space and switches transport of proteins in the space from diffusion to bulk fluid flow [2,11]. Thus, depolymerization of HA in the SC space through the action of hyaluronidases facilitates the dispersion of therapeutics through the SC space and the absorption by the blood and/or lymphatic capillaries, enabling faster and larger volumes of delivery to the subcutaneous space [2,18–21].

Hyaluronidases of animal origin have been used for more than the last 70 years to improve the dispersion and absorption of subcutaneously delivered drugs [22,23]. A recombinant form of human hyaluronidase PH20 (rHuPH20) was developed to lessen allergenicity and immunogenicity concerns associated with animal-derived hyaluronidases, enabling broader therapeutic applications for patients, including repeat-dosing/chronic administration [24–29]. rHuPH20 can be used to optimize SC administration, facilitating faster administration of larger volumes that can reduce the number (and potential frequency) of individual injections required as well as increasing delivery flow rates compared with standard SC administration [12,29,30]. Additionally, use of rHuPH20 can also support the transition of intravenously administered therapies to be given subcutaneously while maintaining the same dosing frequency [29]. At present, rHuPH20 is approved for use as hyaluronidase human injection (Hylenex^®^); as an SC injection for achieving hydration, increasing the dispersion and absorption of other therapeutics; and for SC urography [31]. In addition, rHuPH20 is approved for co-administration with human immunoglobulin for the treatment of primary immunodeficiency (HYQVIA^®^/HyQvia^®^) [30,32]; as a co-formulation with trastuzumab (Herceptin^®^ SC/Herceptin Hylecta™) or pertuzumab and trastuzumab (PHESGO™) for the treatment of human epidermal growth factor receptor 2-positive breast cancer [33–36]; as a co-formulation with daratumumab (DARZALEX FASPRO™/DARZALEX SC) for the treatment of multiple myeloma [37,38]; and as a co-formulation with rituximab for the treatment of chronic lymphocytic leukemia (Rituxan^®^ SC, Rituxan Hycela) and some subtypes of non-Hodgkin lymphoma (MabThera^®^ SC/Rituxan^®^ SC/Rituxan Hycela^TM^) [39–42].

The aim of the current investigation was to characterize the transient and local activity of rHuPH20. Experiments were conducted using an intradermal (ID) route of administration in mice to accommodate anatomical differences of the hypodermis between humans and scruff animals, thereby more closely replicating the local interstitial tissue pressure found within the tighter SC space in humans [43,44]. Concentrations of rHuPH20 equivalent to or above 2000 U/mL were used as they are clinically relevant and approved for use in drug combinations in humans [45]. This investigation comprised two studies: a focused biodistribution study to determine rHuPH20 activity in local skin, regional lymph nodes, and plasma; and a dye dispersion study to assess the pharmacodynamics of systemic rHuPH20 and the impact of local skin administration of rHuPH20 on dispersion in tissue at a distal skin site.

## Methods

### Mouse biodistribution study

#### Test article

The test article contained rHuPH20 (Halozyme Therapeutics Inc., San Diego, CA) at a concentration of 2000 U/mL formulated in histidine buffer (10 mM histidine [Ajinomoto, Raleigh, NC], 130 mM sodium chloride [JT Baker, Phillipsburg, NJ], and 1 mg/mL human serum albumin [Baxter, Deerfield, IL], pH 6.5). rHuPH20 was internally manufactured by Halozyme Therapeutics Inc. with a purity of 99%.

#### Experimental design

All animal studies were conducted in compliance with the National Research Council’s “Guide for the Care and Use of Laboratory Animals” and performed following detailed written protocols approved by Halozyme’s Institutional Animal Care and Use Committee (IACUC, Halozyme protocol number P08123). Female juvenile NCr nu/nu mice (within 8 to 10 weeks of age; Taconic Farms, Hudson, NY) were divided into three groups: ID administration group, systemic exposure group, and untreated control group. Female mice were chosen due to their more docile, less aggressive nature in comparison with male mice. Five animals were assigned to each group for each time point to account for animal-to-animal variation and to establish statistical robustness. Mice were group-housed in plastic rodent cages with filter top lids. Food and water were provided *ad libitum*. Cage bedding was changed daily, the housing room environment was set to maintain a temperature of ~17–27°C and a relative humidity of 40–70%, with a 12-hour light/12-hour dark time cycle. Mice were acclimated to the facility for one week prior to study onset. Mice in the ID administration group were anesthetized by an intraperitoneal injection of ketamine 150 mg/kg (Fort Dodge Animal Health, Fort Dodge, IA) and xylazine 10 mg/kg (Fort Dodge Animal Health, Fort Dodge, IA) diluted in sterile phosphate-buffered saline (Mediatech, Manassas, VA). The mice then received a single 40 μL ID injection of rHuPH20 at 2000 U/mL (80 U total dose) into the left lateral medial aspect. Mice in the systemic exposure group were placed into a restraint device without the use of anesthesia and received a 40 μL injection of rHuPH20 at 2000 U/mL (80 U total dose) intravenously by bolus tail vein injection. Following injection, mice were returned to their housing cage, where they were monitored twice daily (AM and PM). Daily health assessment was taken following IACUC protocol, with body weights taken three times per week until scheduled euthanasia and collection of blood/tissue samples. Mice in the untreated control group remained untreated and served as assay background matrix controls.

#### Tissue and plasma collection

Plasma, lymph nodes on the side of the injection (superficial cervical, axillary, brachial, and inguinal), and skin samples were acquired from the ID administration group and the untreated control group. Samples were collected at 1, 15, 30, 60, 240, 480, and 1440 minutes post-dosing from mice in the ID administration group, and at a single time point from mice in the untreated control group. In the systemic exposure group, plasma alone was acquired at 1, 15, 30, 60, and 240 minutes post-dosing. All plasma samples were collected within 1.8 minutes from the nominal time. For the 1-minute nominal time point, the mean (standard deviation) collection time was 1.97 (0.43) minutes in the ID administration group and 1.33 (0.21) minutes in the systemic exposure group. Lymph nodes and skin samples were collected immediately following plasma sample collection as described below.

At the time of blood/tissue collection, animals were anesthetized with 3–5% isoflurane gas (Minrad, Bethlehem, PA) at 1 L/minute, or no anesthesia if the animal was still sedated from the original anesthesia (ID administration group). Blood was subsequently collected from the sedated animal via cardiac puncture until complete exsanguination and samples were placed into prechilled potassium K_2_-ethylenediaminetetraacetic acid (EDTA)-coated blood collection vials (Becton Dickinson, Franklin Lakes, NJ). Following blood collection, an 8 mm skin biopsy punch (Acuderm, Ft. Lauderdale, FL) was placed directly over the site of injection (ID administration group), or over the left lateral medial aspect (untreated control group), and a full thickness skin sample was collected. Tissue samples were placed into prechilled Lysing Matrix D^®^ tubes (MP Biomedicals, Solon, OH) containing 500 μL lysis buffer (50 mM Tris-hydrochloride [USB, Cleveland, OH], 150 mM sodium chloride [Promega, Madison, WI], 0.1% Igepal CA-630 [Sigma, St. Louis, MO], 1% Triton X-100 [Sigma, St. Louis, MO], and Complete^TM^ Protease Inhibitor Cocktail; one tablet per mL [Roche Applied Science, Indianapolis, IN]). All tubes were snap frozen in liquid nitrogen and stored at –80°C. Frozen tubes were thawed on wet ice and tissues were homogenized using a FastPrep^®^-24 instrument (MP Biomedicals, Solon, OH) at a speed of 5 m/s for 20 seconds for three cycles. Homogenates were centrifuged at 7000 x *g* for 10 minutes at 4°C, and the supernatant collected into prechilled tubes and stored at –80°C until assay.

#### Mouse plasma separation

Cold blood collection tubes containing mouse whole blood and K_2_-EDTA were centrifuged at 3500 x g for 5 minutes at 4°C. The upper plasma layer was collected and transferred to prechilled vials and stored at –80°C until assay.

#### rHuPH20 hyaluronidase activity assay

The rHuPH20 enzyme activity assay was a modification of the method described by Frost et al. [46] and is a measure of hyaluronidase activity in plasma or tissue homogenates. Biotinylated HA substrate (Halozyme Therapeutics, Inc., San Diego, CA) was coated to plastic microtiter plates (Thermo Scientific, Rochester, NY) for 48 hours at 4°C. Diluted plasma and tissue homogenate samples, rHuPH20 calibrators, and controls were incubated in biotinylated HA-coated plates for approximately 90 minutes at 37°C. Plates were washed in phosphate buffered saline/polysorbate 20, and the remaining bound biotinylated HA was detected by the addition of streptavidin-horseradish peroxidase conjugate (Jackson ImmunoResearch Laboratories, West Grove, PA) for 60 minutes, followed by development with the chromogenic substrate tetramethylbenzidine (KPL, Gaithersburg, MD). The optical density (450 nm) value is inversely proportional to the concentration of rHuPH20 in each specimen.

#### Calculations and statistical methods

Data acquisition and analysis were performed using a SpectraMax M2 microplate spectrophotometer and SoftMax Pro v5.1 software (Molecular Devices, Sunnyvale, CA). Concentrations of rHuPH20 in specimens were determined by interpolation from the corresponding standard curve using a non-weighted four-parameter logistic fit. Results within the limits of the standard curve were reported in units per milliliter (U/mL), whereas those below the lower limit of quantitation (LLOQ) were reported as less than the product of the LLOQ and the corresponding sample dilution factor; in this study, values lower than the LLOQ were plotted as 0. For assessment of rHuPH20 activity in plasma and tissue homogenates, assay calibrators were prepared in the corresponding concentration of plasma or tissue homogenate (eg, 1%, 2%, or 4%). Activity in tissue homogenates was also reported as a function of the mass of the tissue sample (ie, in units per milligram of tissue wet weight [U/mg tissue]).

Summary statistics were computed using EXCEL (Microsoft, Seattle, WA) and GraphPad Prism (GraphPad Software, La Jolla, CA).

### Mouse dye dispersion study

#### Test and control articles

Test articles were prepared by half-log serial dilutions of rHuPH20 in diluent ranging from 30 000 U/mL down to a final concentration of 1 U/mL immediately prior to use. Vehicle control animals received diluent alone.

#### Dye dispersion mouse model validation

The sensitivity of the functional dye dispersion mouse model [18] for the impact of systemically delivered hyaluronidase on local skin dispersion (IV dosing) was evaluated in female juvenile NCr nu/nu mice aged within 6 to 8 weeks old. The NCr nu/nu mouse was selected to enable visualization of intradermally delivered dye as an endpoint for dispersion. Mice were restrained and received a 100 μL IV injection into the tail vein of vehicle or rHuPH20 at doses ranging from 0.1 to 3000 U (n = 3 in each dose group; S1 Table). Approximately 30 minutes following injection of vehicle or rHuPH20, animals were anesthetized with an intraperitoneal injection of ketamine 150 mg/kg and xylazine 10 mg/kg to keep them stable during the ID dye dispersion portion of the study. Then, at 45 minutes following IV administration of vehicle or rHuPH20, mice were given two 40 μL ID injections of 0.4% Trypan Blue dye (Gibco, Grand Island, NY), one on each contralateral region frontal of the hind flanks. Dye injections were given 45 minutes after IV administration of vehicle or rHuPH20 based on work by Bookbinder et al. showing that dye dispersion is markedly increased between 30 and 60 minutes following injection of rHuPH20 [18]. Dye dispersion was measured using a digital caliper 5 and 15 minutes after injection of the dye as described previously [18]. Measurements of each dye area were taken in duplicate.

#### Dye dispersion experimental design

The impact of rHuPH20 local ID delivery on dye dispersion at a distal skin site was assessed in female NCr nu/nu mice aged 6 to 8 weeks old. Mice were given a 40 μL ID injection of vehicle or rHuPH20 (dose range from 0.04 to 1200 U; S2 Table) on the right flank while under isoflurane anesthesia. Approximately 30 minutes following injection of vehicle or rHuPH20, animals were given an intraperitoneal injection of ketamine 150 mg/kg and xylazine 10 mg/kg to maintain anesthesia during the ID dye dispersion portion of the study. Mice were then given a 40 μL ID injection of 0.4% Trypan Blue dye on the left side. Dye dispersion was measured using a digital caliper 5 and 15 minutes post-ID injection of the dye, as described previously [18]. Measurements of each dye area were also taken in duplicate.

#### Calculations and statistical methods

The longest diameter of the dye area (length) and the longest diameter perpendicular to the length (width) were taken. The dye area was calculated using the formula for the area of an oval (length x width x 0.8 = length x width x 0.25 π). This formula was selected to most accurately represent the distribution of fluids injected from a beveled needle inserted at an approximately 10-degree angle into the dermis. Statistical significance of dye dispersion area between the vehicle control and rHuPH20 groups were computed using GraphPad Prism (GraphPad Software, San Diego, CA) and determined to be *P*≤0.05 using an analysis of variance (ANOVA) with a Tukey’s multiple comparisons test.

## Results

### Mouse biodistribution study

**Fig 1:**
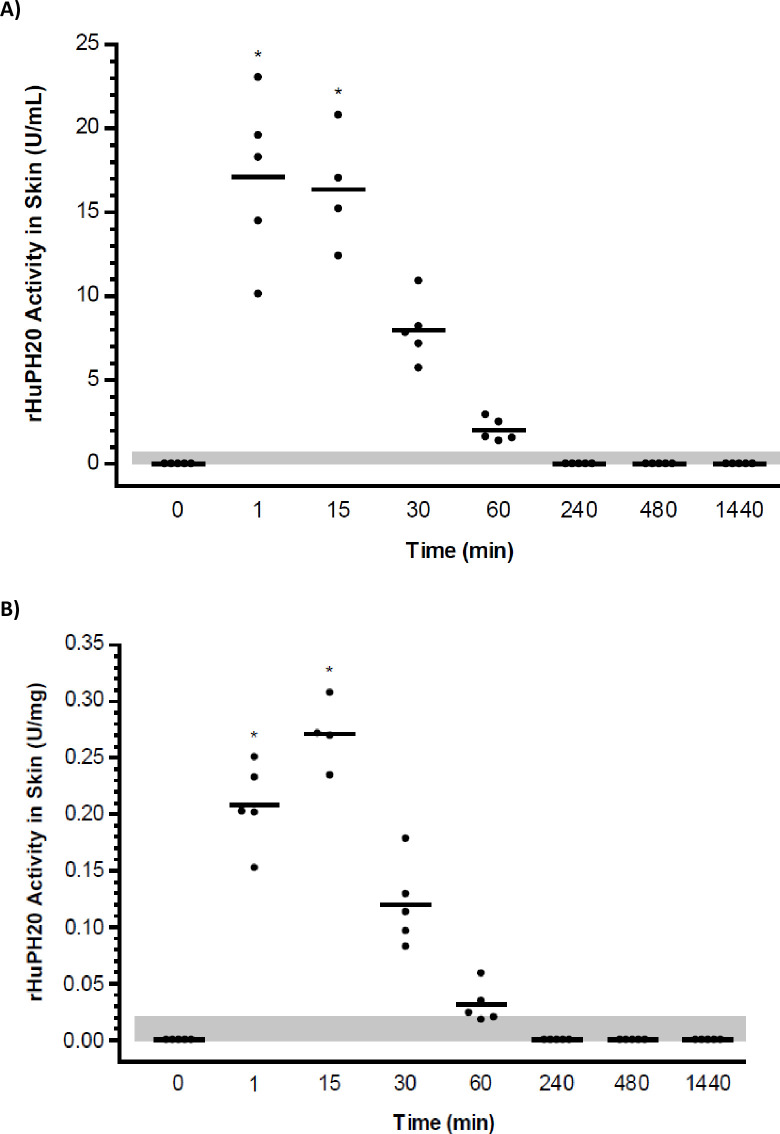
Biodistribution study: Concentration-time course of rHuPH20 in skin samples after ID delivery. rHuPH20 concentration in skin samples from individual animals (circles) are depicted as well as the mean (line) for each time point. (A) Concentration in U/mL, LLOQ = 0.70 U/mL. (B) Concentration in U/mg wet tissue weight, LLOQ = 0.02 U/mg, *P<0.05; two-tailed t-test; samples beyond 15 minutes compared with samples at 1 and 15 minutes. Values lower than the LLOQ (shaded area) are plotted as 0. LOQ, lower limit of quantitation; rHuPH20, recombinant human hyaluronidase PH20.

The biodistribution study assessed rHuPH20 activity in skin, lymph, and plasma after ID administration of rHuPH20 (80 U). In skin samples from the ID injection site, rHuPH20 activity was observed in most tissue homogenates between 1 minute post-dose through to 60 minutes post-dose. The maximum concentration observed in skin homogenates occurred at 1 minute post-dose and ranged from 10.1 to 23.0 U/mL (Fig 1A) or 0.15 to 0.31 U/mg tissue (Fig 1B). Maximum levels of rHuPH20 were observed from 1 to 15 minutes post-dose and declined thereafter. The mean rHuPH20 concentrations (U/mL) for the 1- and 15-minute samples were not statistically different from each other (*P* = 0.81; two-tailed t-test; Fig 1A). However, the mean concentrations for the 1- and 15-minute samples were significantly higher than the values at times beyond 15 minutes for both U/mL (*P*<0.05; two-tailed t-test; Fig 1A) and U/mg (*P*<0.05; two-tailed t-test; Fig 1) measurements. No hyaluronidase activity was detected in any of the skin samples collected from 4 hours through to 24 hours post-dose.

**Fig 2:**
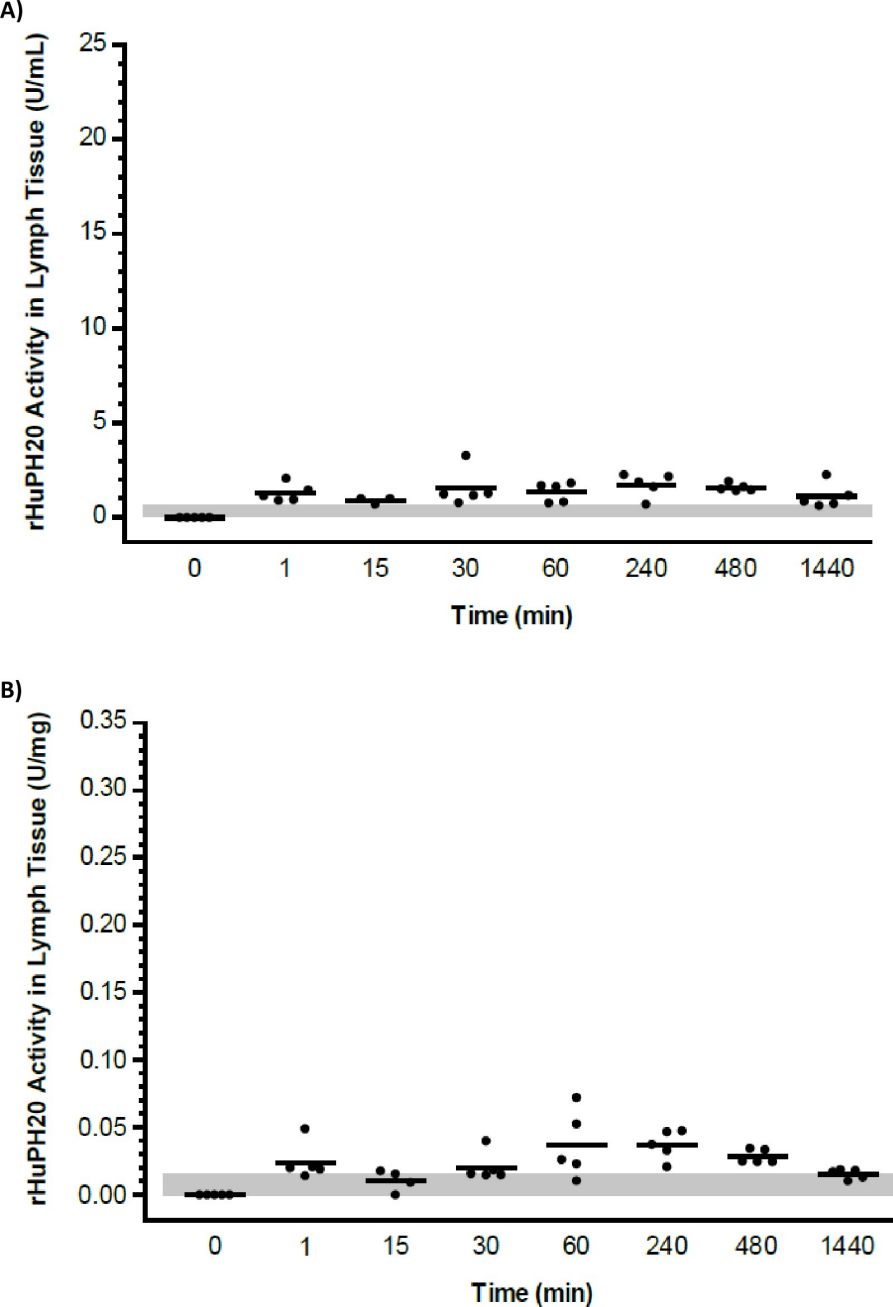
Biodistribution study: Concentration-time course of rHuPH20 in lymph samples after ID delivery. rHuPH20 concentration in lymph samples from individual animals (circles) are depicted as well as the mean (line) for each time point. (A) Concentrations in U/mL, LLOQ = 0.70 U/mL. (B) Concentrations in U/mg wet tissue weight, LLOQ = 0.02 U/mg. Values lower than the LLOQ (shaded area) are plotted as 0. ID, intradermal; LLOQ, lower limit of quantitation; rHuPH20, recombinant human hyaluronidase PH20.

In regional lymph samples, low-level rHuPH20 activity near the detection level was observed in most tissue homogenates between 1 minute post-dose through to 24 hours following ID administration of rHuPH20 (Fig 2). The concentration of rHuPH20 observed in lymph homogenates ranged from 0.65 to 3.3 U/mL (Fig 2A) or below 0.01 to 0.07 U/mg (Fig 2B). As the variability between specimens within a time point exceeded those between time points, no discernible pattern of activity was observed as a function of time after rHuPH20 dosing.

**Fig 3:**
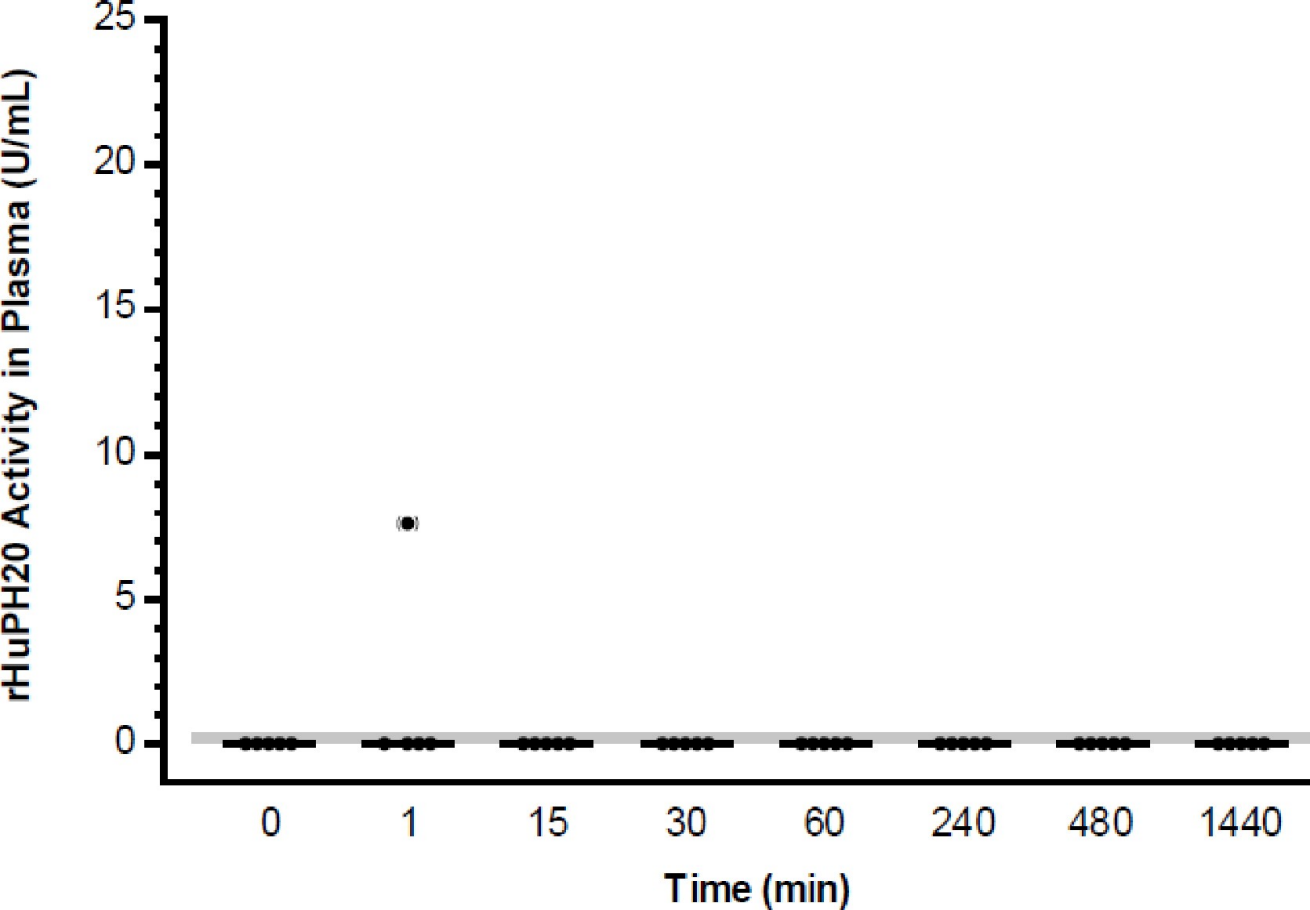
Biodistribution study: Concentration-time course of rHuPH20 in plasma samples after ID delivery. rHuPH20 concentration (U/mL) in plasma samples from individual animals (filled circles) are depicted as well as the mean (line) for each time point. Values lower than the LLOQ (<0.3 U/mL; shaded area) are plotted as 0. The outlier value in parentheses is suspected to have resulted from partial injection of rHuPH20 into the blood compartment and was therefore omitted from the statistical calculations. ID, intradermal; LLOQ, lower limit of quantitation; rHuPH20, recombinant human hyaluronidase PH20.

Hyaluronidase activity was not detected in plasma from 39 of the 40 animals that received an ID injection of 80 U (2000 U/mL) rHuPH20 (Fig 3). A single plasma sample, which was prepared from whole blood collected 2.3 minutes after dosing, revealed a measurable level of rHuPH20 activity of 7.6 U/mL. As the only plasma sample from any ID administered animal with measurable rHuPH20 activity, it is suspected that some of the test article could have been inadvertently injected into the blood compartment. Based on these findings, there was no consistent evidence for systemic exposure after ID delivery of 80 U (2000 U/mL) rHuPH20.

**Fig 4:**
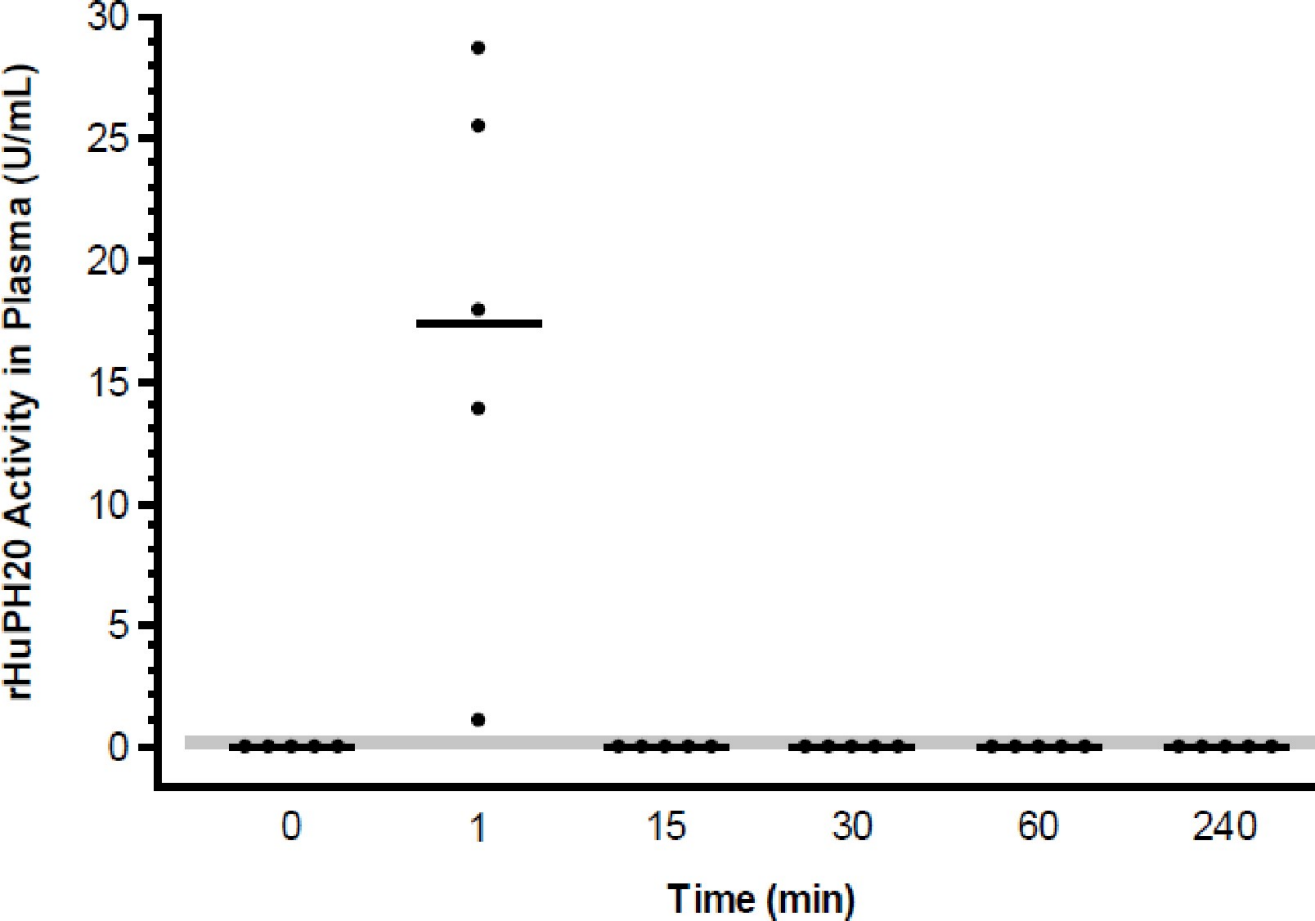
Concentration-time course of rHuPH20 in plasma samples following IV administration. rHuPH20 concentration (U/mL) in plasma samples from individual animals (filled circles) are depicted as well as the mean (line) for each time point. Values lower than the LLOQ (<0.3 U/mL; shaded area) are plotted as 0. IV, intravenous; rHuPH20, recombinant human hyaluronidase PH20; LLOQ, lower limit of quantitation.

In animals who were administered rHuPH20 intravenously (systemic exposure group), rHuPH20 activity was detected in four of the five plasma samples collected 1 minute after dosing (Fig 4). Mean and median values for these four samples were 17.4 and 17.9 U/mL, respectively. The level of rHuPH20 activity in the fifth plasma sample at the same time point was 1.1 U/mL, near the LLOQ of the assay (0.3 U/mL). At any other time point (15 minutes and thereafter), the activity of rHuPH20 could not be detected in plasma. In specimens from untreated animals (untreated control group; plasma, skin, and lymph), all reported enzyme activity values were <0.7 U/mL and thus below the LLOQ for the assay.

### Mouse dye dispersion study

#### Systemic exposure impact on dye dispersion assay

**Fig 5:**
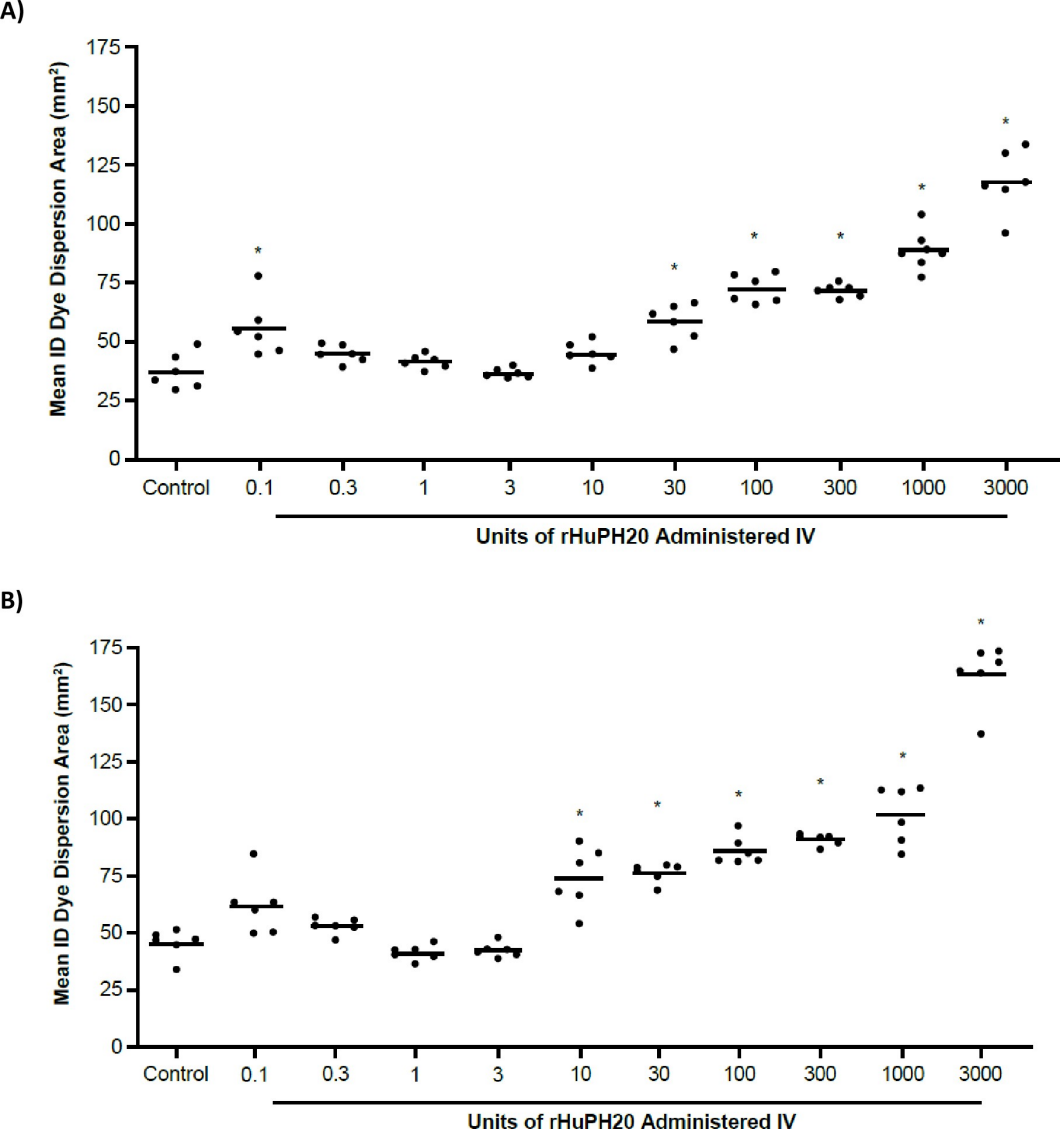
Dye dispersion assay validation: Effect of ascending systemically administered doses of rHuPH20 on intradermal dye dispersion. Measurement of dispersion area (A) 5 minutes and (B) 15 minutes post-dye injection in the skin. Each point represents the mean of duplicate measurements from one animal; the bar represents the mean for all animals. Asterisk denotes statistical significance of *P*<0.05 versus control. IV, intravenously; rHuPH20, recombinant human hyaluronidase PH20.

The sensitivity of the peripheral SC/ID compartment to systemic exposure of rHuPH20 was evaluated by injecting mice intravenously with vehicle control or ascending doses of rHuPH20 (from 0.1 to 3000 U) and, after allowing 45 minutes for rHuPH20 to distribute to the periphery. Trypan Blue dye was injected intradermally into their flanks. In the animals injected intravenously with vehicle control, five minutes after dye injection, the mean area of dye dispersion was 37.0 mm^2^ (Fig 5A). This represented dye dispersion by diffusive properties without the introduction of any rHuPH20.

After IV injection of rHuPH20, a subsequent injection of Trypan Blue in the skin showed a dose-dependent trend of increasing local dye dispersion area as intravenously administered rHuPH20 concentrations increased. At the lower doses of 0.1 to 10 U of rHuPH20, mean dye dispersion areas ranged from 36.1 to 55.3 mm^2^, with no trend for increased dispersion areas with increasing rHuPH20 doses. Doses of rHuPH20 from 30 to 3000 U were statistically different from vehicle control (*P*<0.05, ANOVA) and showed a trend for increasing dispersion area with rHuPH20 dose, resulting in a mean 2.0-fold increase across this range (Fig 5A). Results showed that the administration of 3000 U (the maximum dose of rHuPH20 delivered intravenously) resulted in a 3.2-fold greater dye dispersion area compared with the IV vehicle-injected control mean dye area. Although the mean area of dye dispersion in animals injected with 0.1 U of rHuPH20 was found to be statistically different from the control cohort, one out of the six injections resulted in an unusually large dye dispersion area.

Animals were again measured for dye dispersion area 15 minutes after the initial dye injection in the skin (60 minutes post-IV rHuPH20 administration). At this time point the vehicle control group had a mean dye area of 45.6 mm^2^ (Fig 5B), demonstrating an average normal dispersion increase of 8.6 mm^2^ from the 5-minute time point without rHuPH20 (37.0 to 45.6 mm^2^). The mean dye dispersion areas for animals injected with rHuPH20 doses between with 0.1 U and 3 U ranged between 41.4 to 62.1 mm^2^, which is similar to control (45.6 mm^2^). At higher doses of rHuPH20, mean dye dispersion areas showed a trend for increase with dose when compared with control. The mean dye dispersion areas with rHuPH20 doses between 10 U and 3000 U were approximately 2.2-fold greater than control, and dispersion at the highest rHuPH20 dose was 3.6-fold greater than control. Mean dye dispersion areas ranged from 42.7 mm^2^ at 3 U, to 163.7 mm^2^ at 3000 U. At doses of 10 U or greater of rHuPH20, significantly greater dye dispersion areas (*P*<0.05) were observed when compared with the vehicle control group at 15 minutes post-dye injection, as determined by ANOVA.

#### Impact of locally delivered rHuPH20 on dye dispersion at a distal skin site

**Fig 6:**
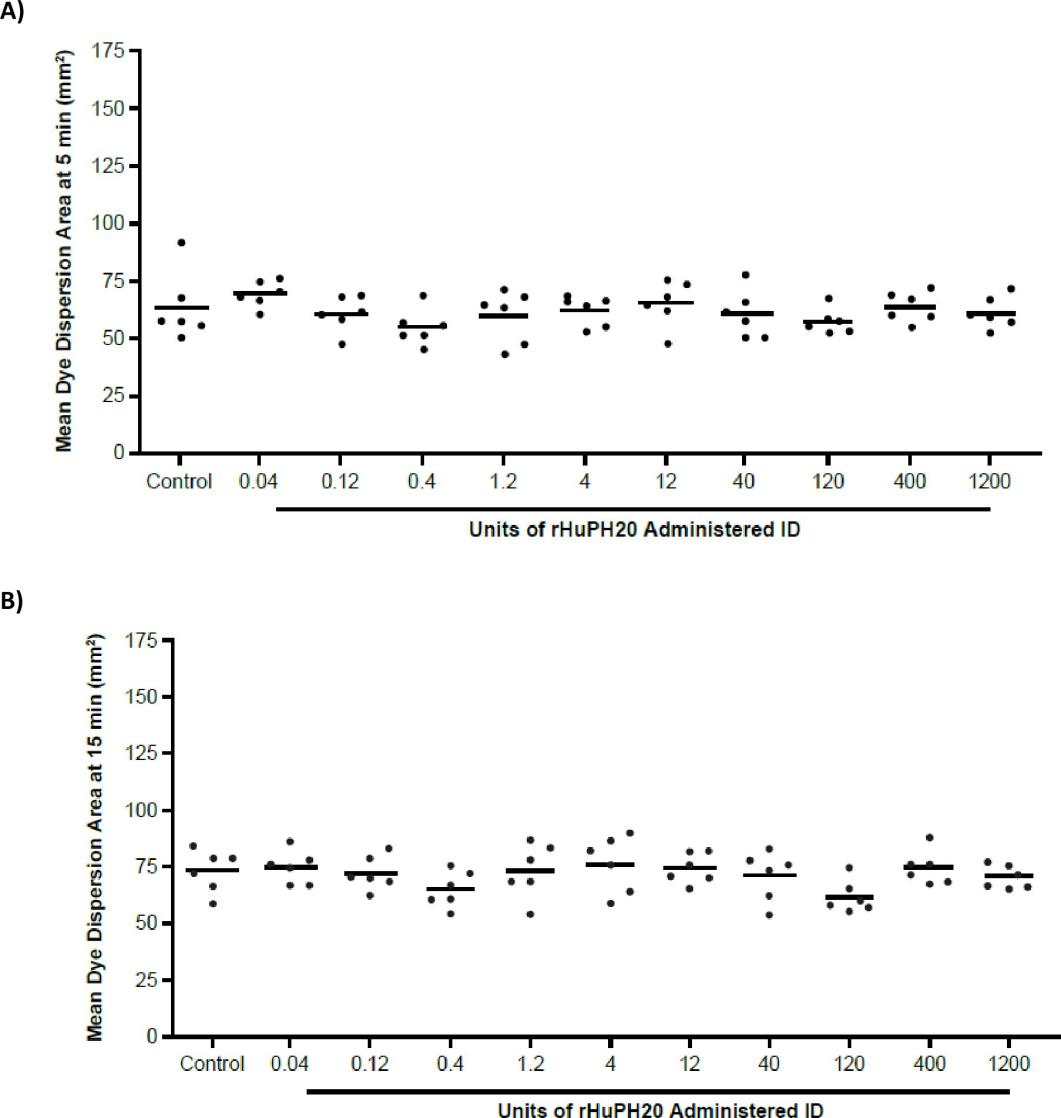
Dye dispersion: Characterization of dispersive effects at a distal site after ascending intradermal rHuPH20 dose administration at a contralateral skin site. Measurement of dye dispersion area (A) 5 minutes and (B) 15 minutes post-dye injection. Each point represents the mean of duplicate measurements from one animal; the bar represents the mean for all animals. ID, intradermally; rHuPH20, recombinant human hyaluronidase PH20.

To evaluate the impact of ID rHuPH20 on dye dispersion at a distal site, mice were injected intradermally on one flank site with vehicle control or ascending doses of rHuPH20 (from 0.04 to 1,200 U) and then, after 45 minutes, injected intradermally with Trypan Blue dye into their contralateral flanks. In the vehicle control group, the mean area of dye dispersion was 63.9 mm^2^ when measured 5 minutes after ID dye injection on the contralateral side (Fig 6A). Mice with intradermally delivered rHuPH20 on one flank did not show an increase in dye dispersion area on the contralateral side over the 4-log dose escalation of rHuPH20. Specifically, all 10 doses of rHuPH20 (from 0.04 to 1200 U) resulted in similar dye dispersion areas on the contralateral sites (with minimum and maximum dispersion areas of 55.5 to 69.7 mm^2^) when compared with the vehicle control group (*P*>0.05, ANOVA).

When measuring dye dispersion areas again at 15 minutes after the dye injection, the vehicle control group had a mean dye dispersion area of 73.2 mm^2^ (Fig 6B). All 10 doses of rHuPH20 (from 0.04 to 1200 U) resulted in similar dye dispersion at a distal skin site compared with vehicle control (*P*>0.05; ANOVA). Overall, the mean dye dispersion area for all rHuPH20 test groups ranged between 61.6 and 76.2 mm^2^. No significant difference in dye dispersion area was observed between the control group and any of the rHuPH20 test groups both at 5 and 15 minutes post-dye injection as determined by ANOVA.

The mean dye dispersion area for all data points increased approximately 15% from the 5- to the 15-minute time point measurement. This increase in overall mean dye area was statistically significant (*P*<0.05; unpaired t-test). The small molecular size of the dye enables diffusion into the ID space, therefore the increase in mean dye area over time is likely due to continuous passive diffusion of the dye.

## Discussion

Findings from the biodistribution and dye dispersion studies presented here further characterize rHuPH20 as a transiently active and locally acting tissue permeation enhancer. The biodistribution study confirmed that ID injection of rHuPH20 has a short half-life in skin over the range of doses studied. The results showed a maximum activity of rHuPH20 from 1 to 15 minutes after dosing; the activity of rHuPH20 declined afterwards. After 4 or more hours after dosing no rHuPH20 activity was detected. Similarly, no discernible pattern of rHuPH20 activity in lymph tissue was identified as a function of time after dosing, as the variability between specimens within a time point was similar to or exceeded those between time points. In general, proteins from the injection site are drained from the interstitial space of the SC tissue to the blood and/or lymphatic capillaries before being absorbed into the systemic circulation [47,48]. The presence of low-level hyaluronidase activity in lymph tissue may therefore suggest that the primary draining lymph nodes are a site of rHuPH20 degradation. Plasma hyaluronidase activity was not detected in the animals that received an ID injection of rHuPH20, with the exception of one animal suspected to have accidentally received a partial dose of rHuPH20 directly into the blood compartment. Overall, these findings suggest that rHuPH20 is either cleared, degraded, or rendered inactive at the local injection site shortly following ID injection and is therefore unlikely to affect HA re-synthesis and the re-establishment of the interstitial matrix as part of normal homeostatic processes. In addition, following IV administration of rHuPH20, enzymatic activity was detected in plasma samples at 1 minute following injection but not at 15 minutes or thereafter, indicating rapid elimination of rHuPH20 from the bloodstream. Overall, these studies demonstrate the local and transient activity of rHuPH20 at concentrations equal to or greater than those used in humans [8,49,50].

The mouse dye dispersion model was found to be functionally sensitive to the effects of systemic levels of rHuPH20 on the dispersion of Trypan Blue dye at a distal skin site. We demonstrated a dose-dependent trend of increasing dye dispersion area as doses of rHuPH20 administered IV increased. Using this model, we demonstrated that ID administration of rHuPH20 did not significantly increase mean dye dispersion at a distal skin site over the dose range studied. Together, these data demonstrate the sensitivity of the model to detect systemic rHuPH20 exposure on the local functional dispersion assay in this mouse model, and establish that a local ID injection of rHuPH20 does not lead to systemic concentrations at a level that would induce functional dispersion at a distal site.

It is important to consider the similarities and differences between human and mice skin when considering human SC delivery based on a rodent model. Mammalian skin is divided into the superficial epidermis, the densely fibrous dermis, and the underlying fatty subcutis, which is the target skin compartment for SC administration in humans [44,51]. In scruff animals, such as rodents, the SC tissue architecture is organized differently, forming a looser skin attachment when compared with human skin [43,44]. Therefore, when rHuPH20 is injected into the tighter human SC space, the rapid depolymerization of HA enables the increased dispersion and absorption of fluids containing therapeutic agents [11]. Scruff animals have a thin dermis layer with low dispersion pressure, limiting the volume that can be delivered. However, in this study, rHuPH20 was delivered at 2000 U/mL, which is the current standard concentration for all co-formulated approved products [36,37,40,52]. It is therefore possible that the distribution of rHuPH20 was not fully captured, for example, if rHuPH20 was dispersed outside of the punch area or was gradually transported via lymph at levels below the LLOQ of the assay. Further studies are thus warranted to characterize the degradation of rHuPH20 at the injection site and in the lymph nodes.

The findings from the studies described here complement previously reported studies suggesting that rHuPH20 acts as a local and transiently active permeation enhancer for the SC administration of therapeutic agents. The short half-life of rHuPH20 following IV injection is in agreement with the findings for animal-derived hyaluronidase. Animal-derived hyaluronidase administrated intravenously was rapidly eliminated from the central compartment when administered to dogs (500 U/kg), humans (500 U/kg), and rats (5000 U/kg), with an elimination half-life of less than 10 minutes [53]. In addition, 10 000 or 30 000 U of rHuPH20 has been administered intravenously to healthy subjects, and the half-life was found to be approximately 10 minutes [54]. Bookbinder et al. [18] administered an ID injection of either 5 U of rHuPH20 or buffer (vehicle) control to NCr nu/nu mice, followed by Trypan Blue dye to the same ID site at different time points from 30 minutes to 48 hours after injection of rHuPH20 or buffer. At 30 and 60 minutes following injection of rHuPH20, the dye dispersion area was markedly increased compared with the control animals that received vehicle alone. Importantly, 24 hours post-rHuPH20 injection the dye dispersion area was comparable with that in the vehicle control animals, demonstrating reconstitution of the local HA barrier and thus confirming the temporary action of the enzyme.

In summary, the current investigation demonstrated that rHuPH20 is a transiently active and locally acting permeation enhancer. These findings complement clinical evidence demonstrating that rHuPH20 can be used to facilitate the dispersion and absorption of subcutaneously delivered therapeutic agents.

## Supporting information

S1 Table rHuPH20 doses in the dye dispersion mouse model validation assay. (DOCX)

S2 Table rHuPH20 doses in the dye dispersion study. (DOCX)
