# Supplementary material for: Dispersive effects and focused biodistribution of recombinant human hyaluronidase PH20: A locally acting and transiently active permeation enhancer
Source: PLoS One. 2021 Jul 22;16(7):e0254765. doi: 10.1371/journal.pone.0254765 (PMC8297837; doi:10.1371/journal.pone.0254765)
Supplement: S1 Table — (DOCX) [file pone.0254765.s001.docx]

**Supplementary Table 1. rHuPH20 doses in the dye dispersion mouse model validation assay**

| rHuPH20 concentration (U/mL) | | Total dose of rHuPH20 (U) |
| --- | --- | --- |
| NA (vehicle) | | NA (vehicle) |
| 1 | 0.1 | |
| 3 | 0.3 | |
| 10 | 1 | |
| 30 | 3 | |
| 100 | 10 | |
| 300 | 30 | |
| 1000 | 100 | |
| 3000 | 300 | |
| 10 000 | 1000 | |
| 30 000 | 3000 | |

IV, intravenous; NA, not applicable; rHuPH20, recombinant human hyaluronidase PH20.
