# Supplementary material for: Dispersive effects and focused biodistribution of recombinant human hyaluronidase PH20: A locally acting and transiently active permeation enhancer
Source: PLoS One. 2021 Jul 22;16(7):e0254765. doi: 10.1371/journal.pone.0254765 (PMC8297837; doi:10.1371/journal.pone.0254765)
Supplement: S2 Table — (DOCX) [file pone.0254765.s002.docx]

**Supplementary Table 2. rHuPH20 doses in the dye dispersion study**

| rHuPH20 concentration (U/mL) | Total dose of rHuPH20 (U) |
| --- | --- |
| NA (vehicle) | NA (vehicle) |
| 1 | 0.04 |
| 3 | 0.12 |
| 10 | 0.4 |
| 30 | 1.2 |
| 100 | 4 |
| 300 | 12 |
| 1000 | 40 |
| 3000 | 120 |
| 10 000 | 400 |
| 30 000 | 1200 |

ID, intradermal; NA, not applicable; rHuPH20, recombinant human hyaluronidase PH20.
